# Factors affecting responsiveness of vadadustat in patients with anemia associated with chronic kidney disease: a post-hoc subgroup analysis of Japanese phase 3 randomized studies

**DOI:** 10.1007/s10157-023-02432-z

**Published:** 2024-03-26

**Authors:** Masaomi Nangaku, Kiichiro Ueta, Kenichi Nishimura, Kazuyo Sasaki, Takafumi Hashimoto

**Affiliations:** 1 Graduate School of Medicine, The University of Tokyo, Tokyo, Japan; 2 Mitsubishi Tanabe Pharma Corporation, Tokyo, Japan

**Keywords:** Anemia, Chronic kidney disease, Hypoxia-inducible factor prolyl hydroxylase inhibitor, Responsiveness, Subgroup analysis, Vadadustat

## Abstract

**Background:**

Vadadustat is an oral hypoxia-inducible factor prolyl hydroxylase inhibitor developed for treating anemia in chronic kidney disease (CKD). The purpose of this post-hoc analysis was to investigate the factors affecting the responsiveness to vadadustat in anemia patients with nondialysis-dependent (NDD) or hemodialysis-dependent (HDD) CKD in two Japanese phase 3 studies.

**Methods:**

Of 151 and 162 patients enrolled in NDD-CKD and HDD-CKD studies, 136 and 140 patients, respectively, were included and divided into subgroups for the analysis. To assess vadadustat responsiveness, the resistance index was defined as the mean body weight-adjusted dose of vadadustat (mg/kg) at weeks 20–24 divided by the mean hemoglobin (g/dL) at weeks 20–24. Multivariate analysis was performed to identify the variables affecting the resistance index.

**Results:**

Independent factors identified as determinants for better response to vadadustat were as follows: high baseline hemoglobin, low baseline eGFR, high week-20–24 ferritin, and CKD not caused by autoimmune disease/glomerulonephritis/vasculitis in NDD-CKD; and male sex, high baseline C-reactive protein, and low baseline erythropoiesis-stimulating agent resistance index (ERI) in HDD-CKD.

**Conclusions:**

In this post-hoc analysis, several factors were identified as affecting the response to vadadustat. These results may provide useful information leading to an appropriate dose modification for vadadustat.

**Clinical trial registration:**

NCT03329196 (MT-6548-J01) and NCT03439137 (MT-6548-J03).

**Supplementary Information:**

The online version contains supplementary material available at 10.1007/s10157-023-02432-z.

## Introduction

Anemia is a common complication of chronic kidney disease (CKD). The frequency and severity of anemia increase as renal function declines [1, 2]. The standard treatment for renal anemia until the recent past has been injectable erythropoiesis-stimulating agents (ESAs), but recently oral hypoxia-inducible factor prolyl hydroxylase (HIF-PH) inhibitors have been approved in Japan, China, Europe, and the United States [3–7]. In addition to these agents, iron supplementation is advised for assistance in maintaining target hemoglobin or to avoid iron deficiency [8].

In Japanese phase 3 studies involving patients with anemia and either nondialysis-dependent (NDD) or hemodialysis-dependent (HDD) CKD, vadadustat, an oral HIF-PH inhibitor, was non-inferior when compared to darbepoetin alfa, an ESA, as evident from the maintenance of hemoglobin levels [9, 10]. In the study of vadadustat in patients with anemia in HDD-CKD, several subgroup analyses carried out on patients selected on the basis of pre-specified patient background on efficacy have also been reported [10]; however, the use of two separate indicators, namely maintenance dose and maintenance hemoglobin levels, both of which vary according to responsiveness, makes interpretation of the results difficult. In the Japanese phase 3 study of vadadustat in anemia patients with NDD-CKD, no subgroup analysis on efficacy has been conducted based on patient background. In Japanese phase 3 studies of roxadustat, another HIF-PH inhibitor, in anemia patients with NDD-CKD or HDD-CKD, only maintenance doses were used to measure efficacy among subgroups known to be related to ESA hyporesponsiveness [11, 12]. In addition, subgroup analyses regarding the efficacy of HIF-PH inhibitors were not adjusted for confounding factors.

This study aimed to investigate the responsiveness to vadadustat in anemia patients with NDD-CKD or HDD-CKD by a post-hoc subgroup analysis of the data from two Japanese phase 3 studies. We analyzed the efficacy of vadadustat on the basis of patient backgrounds using a vadadustat resistance index (VRI), a composite measure of maintenance doses and hemoglobin levels. A multivariate analysis was carried out to identify independent patient background factors affecting responsiveness to vadadustat.

## Materials and methods

### Study design

In a phase 3, open-label, active-controlled noninferiority MT-6548-J01 study (NDD-CKD study, ClinicalTrials.gov, NCT03329196), 304 Japanese adults with anemia in NDD-CKD were randomized to receive either oral vadadustat or subcutaneous darbepoetin alfa for 52 weeks [9]. In another phase 3, double-blind, active-controlled noninferiority MT-6548-J03 study (HDD-CKD study, ClinicalTrials.gov, NCT03439137), 323 Japanese patients who were currently receiving hemodialysis and being treated with ESAs were randomized and switched to either vadadustat or darbepoetin alfa for 52 weeks [10]. These studies were approved by the Institutional Review Boards of each study site and were conducted in compliance with the International Conference on Harmonisation of Technical Requirements for Registration of Pharmaceuticals for Human Use, Good Clinical Practice guidelines, and the Declaration of Helsinki. All individuals voluntarily provided their written informed consent to participate in these studies. Following the agreement based on the contract with Kyowa Kirin Co., Ltd, Tokyo, Japan, the supplier of the control drug darbepoetin alfa, the results of darbepoetin alfa were not included in this post-hoc analysis.

### Study population

In the NDD-CKD study, patients aged ≥ 20 years with CKD who were not on dialysis and had an estimated glomerular filtration rate (eGFR) of < 60 mL/min per 1.73 m^2^ were eligible. Both “ESA users” and “ESA non-users” were included in the study. ESA users had mean hemoglobin levels of 9.0–12.5 g/dL at the last two visits during the screening and had received the same ESA therapy for at least eight weeks before screening. ESA non-users included in the study had mean hemoglobin levels of 8.0–11.0 g/dL and had not received an ESA for eight weeks before screening. During the screening, patients with serum ferritin levels of ≥ 100 ng/mL or transferrin saturation (TSAT) of ≥ 20% were allowed to participate in the study. In the HDD-CKD study, eligible patients were at least 20 years of age; were diagnosed with CKD; underwent either hemodialysis or hemodiafiltration three times a week for ≥ 12 weeks before screening; received the same ESA therapy for at least eight weeks before screening; had a mean hemoglobin level of 9.5–12.0 g/dL, a serum ferritin level of ≥ 100 ng/mL, or a TSAT of ≥ 20%. Details of the inclusion and exclusion criteria in the NDD-CKD and HDD-CKD studies have been reported previously [9, 10].

### Interventions

Starting dose of vadadustat was 300 mg once daily, and doses were adjusted (dose range: 150–600 mg once daily) according to a dose-adjustment algorithm, for maintaining hemoglobin levels within the predefined target ranges of 11.0–13.0 g/dL and 10.0–12.0 g/dL for anemia patients with NDD and HDD-CKD, respectively [9, 10]. These ranges are recommended as treatment targets by the Japanese Society for Dialysis Therapy guidelines [8].

### Endpoints

Considering that the primary endpoint was the mean hemoglobin level at weeks 20–24 of vadadustat treatment in NDD-CKD and HDD-CKD studies [9, 10], the primary endpoint of this post-hoc analysis was set to be equivalent to the VRI at weeks 20–24, stratified by patient backgrounds to assess the vadadustat responsiveness. The VRI was defined as the mean body weight-adjusted dose of vadadustat (dose/kg) at weeks 20–24 divided by the mean hemoglobin (g/dL), referring to the erythropoiesis resistance index [13]. In the HDD-CKD study, dry weight was used as the body weight. The other endpoints included the mean daily dose of vadadustat (mg/day) and mean hemoglobin levels (g/dL) from weeks 20–24, evaluated by the subgroup analysis.

The analysis set for this post-hoc study included patients who had measurement data on “mean dose at weeks 20–24,” “mean hemoglobin level at weeks 20–24,” and “body weight at baseline” and from whom all endpoints were available.

### Statistical analysis

The demographics and characteristics were summarized using descriptive statistics. The mean value (95% confidence interval [CI]) was calculated for the following subgroup categories: age at baseline (< 75, ≥ 75 years old); baseline hemoglobin level (tertile); eGFR (< 15, ≥ 15 to < 30, ≥ 30 mL/min/1.73 m^2^; NDD-CKD study only); duration of dialysis at baseline (< 5, ≥ 5 to < 10, ≥ 10 years; HDD-CKD study only); baseline iron repletion (TSAT ≥ 20% and ferritin ≥ 100 ng/mL, TSAT < 20% and ferritin ≥ 100 ng/mL, TSAT ≥ 20% and ferritin < 100 ng/mL, TSAT < 20% and ferritin < 100 ng/mL); week-20–24 iron repletion (TSAT ≥ 20% and ferritin ≥ 100 ng/mL, TSAT < 20% and ferritin ≥ 100 ng/mL, TSAT ≥ 20% and ferritin < 100 ng/mL, TSAT < 20% and ferritin < 100 ng/mL); baseline ferritin (tertile); week-20–24 ferritin (tertile); baseline TSAT (tertile); week-20–24 TSAT (tertile); presence of diabetes mellitus comorbidity; presence of CKD etiology (diabetes mellitus, hypertension, or autoimmune disease/glomerulonephritis/vasculitis); baseline C-reactive protein (CRP) (< 0.31, ≥ 0.31 mg/dL), baseline erythropoietin resistance index (ERI; tertile, ESA non-user category was set only in the NDD-CKD study); and baseline geriatric nutritional risk index (GNRI) (< 96, ≥ 96 [NDD-CKD study]; < 91.2, ≥ 91.2 [HDD-CKD study]) [14, 15].

Univariate and multivariate analyses were performed to examine the impact of patients' background factors on the primary endpoint. Considering the possibility that the effect of each factor on the VRI may not be linear, the factors observed as continuous variables were classified into 3 or 4 categories for analysis. In univariate analysis, the mean and standard deviation (SD) of the VRI for each factor were calculated, and differences in means between categories were tested and 95% CIs were estimated. For the multivariate analysis, we selected explanatory variables by a forward stepwise method (*p* < 0.05) from factors that were considered general factors (sex, age) and clinically significant (eGFR) as well as those found statistically significant in the univariate analysis of NDD-CKD or HDD-CKD (baseline Hb, week-20–24 ferritin, week-20–24 TSAT, CKD etiology: autoimmune disease/glomerulonephritis/ vasculitis, baseline CRP, and baseline ERI). Week-20–24 iron repletion was not included for the multivariate analysis because it was a composite variable of week-20–24 ferritin and week-20–24 TSAT and highly correlated between them. Then, a multivariate analysis, wherein all explanatory variables are categorical, was performed. In multivariate analysis, the LSmean and standard deviation (SD) of the VRI for each factor were calculated, and differences in LSmeans between categories were tested and 95% CIs were estimated. *P* values of < 0.05 (two-sided) were considered statistically significant.

The reference was set for the upper column category in each factor listed in Tables.

SAS version 9.4 (SAS Institute Inc., Cary, NC, USA) was the software used for all statistical analyses.

## Results

Of 151 and 162 patients in the vadadustat group in the NDD-CKD and HDD-CKD studies, respectively, 15 patients from the NDD-CKD study and 22 patients from the HDD-CKD study were excluded because either they had never received vadadustat, had no efficacy data after randomization, or did not have the data to calculate all endpoints. The remaining 136 and 140 patients, respectively, were included in the analysis set of this post-hoc study, and the VRI was calculated. The demographics and baseline characteristics of NDD-CKD and HDD-CKD patients are shown in Tables 1 and 2, respectively. The mean (SD) age was 71.44 (10.69) years and 65.56 (11.45) years, and the mean baseline hemoglobin level was 10.46 (0.92) g/dL and 10.73 (0.73) g/dL in the NDD-CKD and HDD-CKD studies, respectively. The mean baseline eGFR was 21.78 (11.77) mL/min/1.73 m^2^ in the NDD-CKD study, and the mean duration of dialysis was 7.16 (6.61) years in the HDD-CKD study. The proportion of iron repletion (ferritin ≥ 100 ng/mL and TSAT ≥ 20%) at baseline was 53.7% and 50.0% in the NDD-CKD and HDD-CKD studies, respectively (Tables 4 and 5).

**Table 1:** Patient demographics and characteristics in patients with nondialysis-dependent CKD

Factor	Category	No. of patients (%)	Mean	SD
Age [years]	All	136	71.44	10.69
	< 75	75 (55.15)		
	≥ 75	61 (44.85)		
Sex	Male	66 (48.53)		
	Female	70 (51.47)		
Hb [g/dL], baseline	All	136	10.46	0.92
	≤ 10.0	49 (36.03)		
	> 10.0– ≤ 10.9	43 (31.62)		
	> 10.9	44 (32.35)		
eGFR [mL/min/1.73 m^2^], baseline	All	136	21.78	11.77
	< 15	52 (38.24)		
	≥ 15– < 30	49 (36.03)		
	≥ 30	35 (25.74)		
Ferritin [ng/mL], baseline	All	136	144.54	106.40
	> 162.0	46 (33.82)		
	> 81.1– ≤ 162.0	45 (33.09)		
	≤ 81.1	45 (33.09)		
Ferritin [ng/mL], week 20–24	All	136	105.28	75.43
	> 121.5	46 (33.82)		
	> 66.4– ≤ 121.5	44 (32.35)		
	≤ 66.4	46 (33.82)		
TSAT [%], baseline	All	136	30.89	10.52
	> 35	43 (31.62)		
	> 25– ≤ 35	46 (33.82)		
	≤ 25	47 (34.56)		
TSAT [%], week 20–24	All	136	28.99	9.88
	> 31	46 (33.82)		
	> 24– ≤ 31	43 (31.62)		
	≤ 24	47 (34.56)		
Diabetic comorbidity	No	87 (63.97)		
	Yes	49 (36.03)		
CKD etiology: Diabetes	No	96 (70.59)		
	Yes	40 (29.41)		
CKD etiology: Hypertension	No	90 (66.18)		
	Yes	46 (33.82)		
CKD etiology: AD/GN/Vasculitis	No	118 (86.76)		
	Yes	18 (13.24)		
CRP [mg/dL], baseline	All	136	0.24	0.61
	< 0.31	116 (85.29)		
	≥ 0.31	20 (14.71)		
ERI [IU/kg/week/g/dL], baseline	All	136	5.01	3.60
	≤ 3.16	23 (16.91)		
	> 3.16– ≤ 5.13	23 (16.91)		
	> 5.13	24 (17.65)		
	No Pre ESA	66 (48.53)		
GNRI, baseline	All	136	99.71	9.32
	< 96	51 (37.50)		
	≥ 96	85 (62.50)		

*SD* standard deviation, *Hb* hemoglobin, *eGFR* estimated glomerular filtration rate, *TSAT* transferrin saturation, *AD/DN* autoimmune disease/glomerulonephritis, *CRP* C-reactive protein, *ERI* erythropoiesis-stimulating agent resistance index, *GNRI* geriatric nutritional risk index

**Table 2:** Patient demographics and characteristics in patients with hemodialysis-dependent CKD

Factor	Category	No. of patients (%)	Mean	SD
Age [years]	All	140	65.56	11.45
	< 75	108 (77.14)		
	≥ 75	32 (22.86)		
Sex	Male	92 (65.71)		
	Female	48 (34.29)		
Hb [g/dL], baseline	All	140	10.73	0.73
	≤ 10.3	47 (33.57)		
	> 10.3– ≤ 11.0	46 (32.86)		
	> 11.0	47 (33.57)		
Dialysis period [years]	All	140	7.16	6.61
	< 5	71 (50.71)		
	≥ 5– < 10	33 (23.57)		
	≥ 10	36 (25.71)		
Ferritin [ng/mL], baseline	All	140	147.97	147.84
	> 163.0	47 (33.57)		
	> 82.7– ≤ 163.0	46 (32.86)		
	≤ 82.7	47 (33.57)		
Ferritin [ng/mL], week 20–24	All	140	150.10	173.91
	> 157.0	46 (32.86)		
	> 78.35– ≤ 157.0	47 (33.57)		
	≤ 78. 35	47 (33.57)		
TSAT [%], baseline	All	140	28.28	10.15
	> 30.0	52 (37.14)		
	> 22.0– ≤ 30.0	47 (33.57)		
	≤ 22.0	41 (29.29)		
TSAT [%], week 20–24	All	140	28.78	8.93
	> 31.0	47 (33.57)		
	> 24.0– ≤ 31.0	48 (34.29)		
	≤ 24.0	45 (32.14)		
Diabetic comorbidity	No	110 (78.57)		
	Yes	30 (21.43)		
CKD etiology: diabetes	No	115 (82.14)		
	Yes	25 (17.86)		
CKD etiology: hypertension	No	122 (87.14)		
	Yes	18 (12.86)		
CKD etiology: AD/GN/Vasculitis	No	87 (62.14)		
	Yes	53 (37.86)		
CRP [mg/dL], baseline	All	140	0.28	0.97
	< 0.31	115 (82.14)		
	≥ 0.31	25 (17.86)		
ERI [IU/kg/week/g/dL], baseline	All	140	5.61	3.81
	≤ 3.4818	47 (33.57)		
	> 3.4818– ≤ 6.3429	47 (33.57)		
	> 6.3429	46 (32.86)		
GNRI, baseline	All	140	94.86	8.02
	< 91.2	48 (34.29)		
	≥ 91.2	92 (65.71)		

*SD* standard deviation, *Hb* hemoglobin, *TSAT* transferrin saturation, *AD/DN* autoimmune disease/glomerulonephritis, *CRP* C-reactive protein, *ERI* erythropoiesis-stimulating agent resistance index, *GNRI* geriatric nutritional risk index

Mean and SD of VRI and its components, dose of vadadustat, and body weight in NDD-CKD and HDD-CKD are shown in Table 3. Baseline hemoglobin, week-20–24 iron repletion, week-20–24 ferritin, week-20–24 TSAT, CKD caused by autoimmune disease, glomerulonephritis, or vasculitis and baseline ERI were statistically significant in the univariate analysis performed in the NDD-CKD study (Table 4). The forest plots of the VRI in relation to patient background factors are shown in Supplementary Fig. 1. Multivariate analyses revealed that in patients with NDD-CKD, high baseline hemoglobin, low baseline eGFR, high week-20–24 ferritin, and CKD not due to autoimmune disease/glomerulonephritis/vasculitis were independent factors associated with better vadadustat response (Table 4). In all categories by patient background, mean hemoglobin levels at weeks 20–24 were within the target range (11.0–13.0 g/dL) (Supplementary Table 1). The mean doses at weeks 20–24 in all categories were 282–482 mg/day, as shown in Supplementary Table 1. Irrespective of the baseline eGFR categories, vadadustat treatment decreased serum hepcidin to almost similar levels during the study (Supplementary Table 3). There was no clear difference in baseline CRP levels between patients with and without autoimmune diseases or glomerulonephritis or vasculitis (Supplementary Table 4). Regardless of baseline CRP levels, vadadustat reduced serum hepcidin and maintained it at approximately similar levels (Supplementary Table 5).

**Table 3:** VRI, dose of vadadustat, and body weight in patients with nondialysis-dependent or hemodialysis-dependent-CKD

	NDD-CKD				HDD-CKD			
	*N*	Mean	95% CI	SD	*N*	Mean	95% CI	SD
VRI	136	0.600	0.542, 0.658	0.340	140	0.644	0.582, 0.706	0.370
Mean dose (mg), week 20–24	136	379.26	347.53, 410.98	187.07	140	375.34	343.45, 407.23	190.85
Body weight (kg), baseline	136	57.40	55.34, 59.45	12.11	140	58.66	56.62, 60.70	12.19

*VRI* vadadustat resistance index, *CI* confidence interval, *SD* standard deviation

**Table 4:** Factors associated with the vadadustat resistance index (VRI) in patients with nondialysis-dependent CKD

			Univariate analysis					Multivariate analysis			
		*N*	VRI mean	SD	Difference of mean	95% CI	*p* value	VRI LS mean	Difference of LS mean	95% CI	*p*-value
Age [years]	< 75	75	0.584	0.328	–	–	–				
	≥ 75	61	0.620	0.357	0.035	−0.081, 0.152	0.549				
Sex	Male	66	0.573	0.323	–	–	–				
	Female	70	0.626	0.357	0.053	−0.063, 0.168	0.368				
Hb [g/dL], baseline	≤ 10.0	49	0.721	0.366	–	–	–	0.881	–	–	–
	> 10.0– ≤ 10.9	43	0.520	0.302	−0.201	−0.338, − 0.064	0.004	0.732	−0.149	−0.269, − 0.029	0.015
	> 10.9	44	0.544	0.315	−0.177	−0.313, − 0.042	0.011	0.644	−0.237	−0.364, − 0.110	< 0.001
eGFR[mL/min/1.73m^2^], baseline	< 15	52	0.565	0.314	–	–	–	0.636	–	–	–
	≥ 15 – < 30	49	0.613	0.375	0.047	−0.087, 0.182	0.488	0.760	0.124	0.008, 0.240	0.037
	≥ 30	35	0.635	0.332	0.070	−0.078, 0.218	0.352	0.862	0.226	0.088, 0.364	0.002
Iron repletion (TSAT[%]/Ferritin[ng/mL]), baseline	≥ 20/ ≥ 100	73	0.588	0.324	–	–	–				
	< 20/ ≥ 100	8	0.494	0.330	−0.094	−0.343, 0.155	0.457				
	≥ 20/ < 100	47	0.665	0.377	0.077	−0.048, 0.203	0.224				
	< 20/ < 100	8	0.434	0.203	−0.154	−0.404, 0.095	0.223				
Iron repletion (TSAT[%]/Ferritin[ng/mL]), week 20–24	≥ 20/ ≥ 100	55	0.462	0.323	–	–	–				
	< 20/ ≥ 100	5	0.569	0.312	0.108	−0.189, 0.404	0.474				
	≥ 20/ < 100	62	0.729	0.328	0.267	0.15, 0.384	< 0.001				
	< 20/ < 100	14	0.586	0.274	0.124	−0.066, 0.314	0.198				
Ferritin [ng/mL], baseline	> 162	46	0.563	0.342	−	–	–				
	> 81.1 – ≤ 162.0	45	0.604	0.322	0.041	−0.101, 0.183	0.570				
	≤ 81.1	45	0.635	0.360	0.072	−0.070, 0.214	0.318				
Ferritin [ng/mL], week 20-24	> 121.5	46	0.458	0.322	–	–	–	0.637	–	–	–
	> 66.4– ≤ 121.5	44	0.651	0.379	0.192	0.056, 0.329	0.006	0.784	0.147	0.023, 0.272	0.021
	≤ 66.4	46	0.694	0.274	0.236	0.101, 0.371	< 0.001	0.837	0.201	0.082, 0.320	0.001
TSAT [%], baseline	> 35	43	0.657	0.330	–	–	–				
	> 25– ≤ 35	46	0.620	0.369	−0.037	−0.179, 0.105	0.606				
	≤ 25	47	0.528	0.315	−0.129	−0.270, 0.013	0.074				
TSAT [%], week 20–24	> 31	46	0.529	0.345	–	–	–				
	> 24– ≤ 31	43	0.672	0.356	0.143	0.001, 0.285	0.048				
	≤ 24	47	0.605	0.313	0.076	−0.063, 0.215	0.279				
Diabetic comorbidity	No	87	0.610	0.347	–	–	–				
	Yes	49	0.582	0.331	−0.028	−0.149, 0.093	0.648				
CKD etiology: Diabetes	No	96	0.629	0.351	–	–	–				
	Yes	40	0.531	0.306	− 0.099	−0.225, 0.027	0.124				
CKD etiology: Hypertension	No	90	0.586	0.348	–	–					
	Yes	46	0.629	0.326	0.043	−0.079, 0.165	0.487				
CKD etiology: AD/GN/Vasculitis	No	118	0.561	0.321	–	–	–	0.633	–	–	–
	Yes	18	0.857	0.362	0.296	0.133, 0.459	< 0.001	0.872	0.239	0.085, 0.393	0.003
CRP [mg/dL], baseline	< 0.31	116	0.606	0.338	–	–	–				
	≥ 0.31	20	0.567	0.361	−0.039	−0.203, 0.124	0.637				
ERI [IU/kg/week/g/dL], baseline	≤ 3.16	23	0.565	0.277	–	–	–	0.729	–	–	–
	> 3.16– ≤ 5.13	23	0.706	0.315	0.141	−0.049, 0.331	0.143	0.827	0.098	−0.073, 0.269	0.257
	> 5.13	24	0.789	0.359	0.224	0.036, 0.412	0.020	0.879	0.150	−0.021, 0.322	0.086
	No Pre ESA	66	0.508	0.331	−0.057	−0.213, 0.099	0.470	0.575	−0.154	−0.300, −0.007	0.040
GNRI, baseline	< 96	51	0.667	0.402	–	–	–				
	≥ 96	85	0.560	0.293	−0.107	−0.226, 0.011	0.075				

*VRI* vadadustat resistance index, *CI* confidence interval, *SD* standard deviation, *Hb* hemoglobin, *eGFR* estimated glomerular filtration rate, *TSAT* transferrin saturation, *AD/DN* autoimmune disease/glomerulonephritis, *CRP* C-reactive protein, *ERI* erythropoiesis-stimulating agent resistance index, *GNRI* geriatric nutritional risk index

For the multivariate analysis, we selected explanatory variables by a forward stepwise method (p < 0.05) from factors that were considered general factors (sex, age) and clinically significant (eGFR) as well as those found statistically significant in the univariate analysis of NDD-CKD (baseline Hb, week 20–24 ferritin, week 20–24 TSAT, CKD etiology: AD/GN/Vasculitis, baseline CRP, and baseline ERI). Week 20–24 iron repletion was not included for the multivariate analysis because it was a composite variable of week-20–24 ferritin and week 20–24 TSAT and highly correlated between them. Then, a multivariate analysis was performed. *P* values of < 0.05 (two-sided) were considered statistically significant. The reference was set for the upper column category in each factor listed

In the HDD-CKD study, sex, baseline CRP, and baseline ERI were statistically significant, as observed in univariate analysis (Table 5). The forest plots of the vadadustat resistance indices by patients' background factors are shown in Supplementary Fig. 2. Multivariate analysis identified male, baseline high CRP, and baseline low ERI as independent factors associated with better vadadustat response (Table 5). In all categories by patient background, the mean hemoglobin level at weeks 20–24 was within the target range (10.0–12.0 g/dL) (Supplementary Table 2). The mean doses at weeks 20–24 in all categories were 307–445 mg/day in HDD-CKD studies (Supplementary Table 2). In the HDD-CKD study, there was no clear difference in baseline CRP levels between patients with and without autoimmune diseases or glomerulonephritis or vasculitis (Supplementary Table 4). Like in the NDD-CKD, regardless of whether baseline CRP was high or low, vadadustat lowered serum hepcidin and maintained it at approximately similar levels (Supplementary Table 5).

**Table 5:** Factors associated with the vadadustat resistance index (VRI) in patients with hemodialysis-dependent CKD

		*N*	Univariate analysis					Multivariate analysis			
			VRI mean	SD	Difference of mean	95% CI	*p* value	VRI LS mean	Difference of LS mean	95% CI	*p* value
Age [years]	< 75	108	0.647	0.372	–		–				
	≥ 75	32	0.634	0.369	−0.012	−0.16, 0.135	0.869				
Sex	Male	92	0.545	0.336	–	–	–	0.518	–	–	–
	Female	48	0.834	0.361	0.289	0.168, 0.411	< 0.001	0.730	0.212	0.092, 0.331	< 0.001
Hb [g/dL], baseline	≤ 10.3	47	0.699	0.394	–	–	–				
	> 10.3 – ≤ 11.0	46	0.659	0.360	−0.040	−0.192, 0.111	0.599				
	> 11.0	47	0.574	0.350	−0.126	−0.276, 0.025	0.101				
Dialysis period [years]	< 5	71	0.636	0.340	–	–	–				
	≥ 5 – < 10	33	0.646	0.383	0.011	−0.144, 0.166	0.892				
	≥ 10	36	0.658	0.421	0.022	−0.129, 0.173	0.774				
Iron repletion (TSAT[%]/Ferritin[ng/mL]), baseline	≥ 20/ ≥ 100	70	0.648	0.368	–	–	–				
	< 20/ ≥ 100	16	0.554	0.328	−0.094	− 0.297, 0.110	0.363				
	≥ 20/ < 100	43	0.688	0.387	0.040	−0.102, 0.182	0.580				
	< 20/ < 100	11	0.573	0.386	−0.075	−0.313, 0.163	0.536				
Iron repletion (TSAT[%]/Ferritin[ng/mL]), week 20–24	≥ 20/ ≥ 100	69	0.625	0.335	–	–	–				
	< 20/ ≥ 100	8	0.518	0.503	−0.107	−0.381, 0.166	0.439				
	≥ 20/ < 100	51	0.704	0.408	0.079	−0.056, 0.214	0.251				
	< 20/ < 100	12	0.580	0.287	−0.045	−0.274, 0.184	0.699				
Ferritin [ng/mL], baseline	> 163.0	47	0.620	0.353	–	–	–				
	> 82.7 – ≤ 163.0	46	0.665	0.371	0.044	−0.108, 0.197	0.568				
	≤ 82.7	47	0.647	0.392	0.026	−0.125, 0.178	0.731				
Ferritin [ng/mL], week 20-24	> 157.0	46	0.599	0.358	–	–	–				
	> 78.35 – ≤ 157.0	47	0.646	0.386	0.048	−0.104, 0.200	0.536				
	≤ 78.35	47	0.685	0.368	0.087	−0.065, 0.239	0.261				
TSAT [%], baseline	30.0 <	52	0.690	0.395	–	–	–				
	> 22.0 – ≤ 30.0	47	0.603	0.353	−0.087	−0.235, 0.060	0.245				
	≤ 22.0	41	0.631	0.358	−0.059	−0.212, 0.094	0.449				
TSAT [%], week 20–24	> 31.0	47	0.602	0.372	–	–	–				
	> 24.0 – ≤ 31.0	48	0.681	0.378	0.079	−0.072, 0.230	0.301				
	≤ 24.0	45	0.647	0.363	0.044	−0.109, 0.198	0.567				
Diabetic comorbidity	No	110	0.641	0.374	–	–	–				
	Yes	30	0.655	0.361	0.014	−0.137, 0.165	0.853				
CKD etiology: Diabetes	No	115	0.641	0.374	–	–	–				
	Yes	25	0.658	0.355	0.017	−0.145, 0.179	0.833				
CKD etiology: Hypertension	No	122	0.644	0.376	–	–	–				
	Yes	18	0.643	0.336	0.000	−0.186, 0.185	0.997				
CKD etiology: AD/GN/Vasculitis	No	87	0.604	0.355	–	–	–				
	Yes	53	0.709	0.388	0.105	−0.021, 0.232	0.102				
CRP [mg/dL], baseline	< 0.31	115	0.679	0.364	–	–	–	0.708	–	–	–
	≥ 0.31	25	0.480	0.357	−0.199	−0.358, −0.041	0.014	0.540	−0.168	−0.313, −0.024	0.022
ERI [IU/kg/week/g/dL], baseline	≤ 3.4818	47	0.468	0.325	–	–	–	0.482	–	–	–
	> 3.4818– ≤ 6.3429	47	0.668	0.357	0.199	0.058, 0.341	0.006	0.634	0.153	0.018, 0.287	0.026
	> 6.3429	46	0.798	0.356	0.330	0.188, 0.472	< 0.001	0.755	0.274	0.136, 0.412	< 0.001
GNRI, baseline	< 91.2	48	0.658	0.391	–	–	–				
	≥ 91.2	92	0.636	0.361	−0.022	−0.152, 0.109	0.746				

*VRI* vadadustat resistance index, *SD* standard deviation, *CI* confidence interval, *Hb* hemoglobin, *TSAT* transferrin saturation, *AD/DN* autoimmune disease/glomerulonephritis, *CRP* C-reactive protein, *ERI* erythropoiesis-stimulating agent resistance index, *GNRI* geriatric nutritional risk index

For the multivariate analysis, we selected explanatory variables by a forward stepwise method (p < 0.05) from factors that were considered general factors (sex, age) as well as those found statistically significant in the univariate analysis of HDD-CKD (baseline Hb, week-20–24 ferritin, week-20–24 TSAT, CKD etiology: AD/GN/Vasculitis, baseline CRP, and baseline ERI). Week-20–24 iron repletion was not included for the multivariate analysis because it was a composite variable of week-20–24 ferritin and week-20–24 TSAT and highly correlated between them. Then, a multivariate analysis was performed. *P* values of < 0.05 (two-sided) were considered statistically significant. The reference was set for the upper column category in each factor listed

## Discussion

We evaluated the responsiveness to vadadustat in patients with anemia in NDD-CKD and HDD-CKD patients, stratified by patients' backgrounds, using the VRI in this post-hoc analysis of the Japanese phase 3 studies. Multivariate analyses demonstrated that, as independent factors associated with better vadadustat response, high baseline hemoglobin, low baseline eGFR, high week-20–24 ferritin, and CKD with causes other than autoimmune disease/glomerulonephritis/vasculitis in patients with NDD-CKD; and male sex, high baseline CRP, and low baseline ERI in those with HDD-CKD.

This analysis identified baseline ERI as an independent factor influencing responsiveness in patients with HDD-CKD, with a higher VRI at higher ERIs. Although not identified as an independent factor in NDD-CKD, a similar trend to HDD-CKD was observed. Similar results have been reported in the post-hoc studies of the HIF-PH inhibitor roxadustat in patients with NDD-CKD and HDD-CKD, according to which the maintenance doses of roxadustat and darbepoetin alfa are higher if the baseline ERI is high [11, 12]. Previous studies have reported that iron deficiency, decreased renal function, and inflammation were predictors of ESA hyporesponsiveness [16, 17]. Regarding iron deficiency status, low week-20–24 ferritin levels indicating lower levels of stored iron, negatively influenced responsiveness to vadadustat in NDD-CKD patients in this analysis. However, in patients with HDD-CKD, the VRI tended to increase with decreasing week-20–24 ferritin levels, which was not identified as an independent factor affecting vadadustat responsiveness. Since all patients switched from ESAs, hemoglobin levels were maintained from baseline to 24 weeks following vadadustat administration in patients with HDD-CKD. In contrast, hemoglobin levels were found to increase by approximately 1 g/dL in patients with NDD-CKD, suggesting that the amount of stored iron might have affected vadadustat responsiveness during the hemoglobin increase phase. The present results are consistent with the reports that iron supplementation was associated with a lower ESA dose and an augmented hemoglobin response by HIF-PH inhibitor [11, 12]. Therefore, especially during the hemoglobin elevation phase, appropriate iron supplementation may be essential from the viewpoint of vadadustat responsiveness.

As for renal function, baseline eGFR was identified as an independent factor influencing the vadadustat responsiveness in patients with NDD-CKD, unexpectedly demonstrating a higher VRI at higher eGFR. It is not clear why high baseline eGFR was associated with lowered vadadustat responsiveness. Nevertheless, the vadadustat responsiveness was not attenuated in the low baseline eGFR population, who are assumed to have a reduced ability to produce erythropoietin and mechanistically decreased responsiveness to HIF-PH inhibitor [18]. Similar to our present study, in the post-hoc analysis of the phase 3 study of roxadustat, the maintenance dose of roxadustat for patients with eGFR < 15 mL/min/1.73 m^2^ at baseline was not increased than those with eGFR ≥ 15 mL/min/1.73 m^2^ [11]. In contrast, approximately twice the maintenance dose of darbepoetin alfa was required in the patients with eGFR < 15 mL/min/1.73 m^2^ as opposed to those with eGFR ≥ 15 mL/min/1.73 m^2^ [11], reinforcing the previous results that decreased renal function was associated with hyporesponsiveness to ESA [16]. Serum levels of hepcidin, which are known to negatively regulate iron utilization during hematopoiesis, have been observed to increase with decreased renal function [17]. Serum hepcidin levels in the darbepoetin alfa group transiently decreased with hematopoiesis but returned to baseline levels at 24 weeks, while serum hepcidin levels in the vadadustat group remained decreased after 24 weeks, even though maintained hemoglobin levels between the two groups were comparable in the NDD-CKD study [9]. Although the baseline serum hepcidin levels were higher in patients with low eGFR, vadadustat reduced the serum hepcidin levels and maintained at almost similar levels, irrespective of the baseline eGFR categories (Supplementary Table 3). Taken together, the difference in the effect of HIF-PH inhibitors and darbepoetin alfa on responsiveness in the low eGFR population may partly be attributed to their different effects on serum hepcidin. In the present analysis, the fact that vadadustat responsiveness was not attenuated in patients with eGFR < 15 mL/min/1.73 m^2^, suggests that HIF-PH inhibitors would be a useful treatment option in patients with reduced renal function.

Regarding inflammation status, in patients with HDD-CKD, baseline low CRP levels were independently associated with lower vadadustat responsiveness; however, this association was not observed in the NDD-CKD study. The reason for these unexpected results in HDD-CKD is unclear; however, vadadustat responsiveness was not decreased with high CRP levels. In the post-hoc study of roxadustat in patients with NDD-CKD and HDD-CKD, while the maintenance dose of roxadustat was not affected by baseline high-sensitive (hs)-CRP, the maintenance dose of darbepoetin alfa did show an increasing trend in the high hs-CRP group [11, 12]. Inflammatory indices, such as CRP, are reportedly negatively correlated with ESA responsiveness, and inflammatory cytokines can increase hepatic hepcidin expression, suppressing iron utilization [17]. The difference in the effects of HIF-PH inhibitors and darbepoetin alfa on hepcidin may also contribute to their responsiveness in inflammatory conditions similar to those in the case of reduced renal function. As the responsiveness to vadadustat did not decrease in inflammatory conditions with high CRP levels at baseline, vadadustat may be a better option for treating anemia in patients with inflammation. Because of the small number of patients with CRP ≥ 0.31 mg/dL (about 1/5 of those with CRP < 0.31 mg/dL), further research is needed to conclude the effect of inflammatory status on the responsiveness to vadadustat.

The association between diabetes or gender and ESA hyporesponsiveness is still controversial [16, 19, 20]. In this analysis, diabetic comorbidity and CKD caused by diabetes were not related to VRI in patients with NDD-CKD and HDD-CKD. Among the primary diseases of CKD, autoimmune disease/glomerulonephritis/vasculitis was identified as an independent factor and associated with relatively poor responsiveness to vadadustat in patients with NDD-CKD. However, in patients with HDD-CKD, CKD caused by autoimmune disease/glomerulonephritis/vasculitis did not affect the resistance index. Since the number of patients with CKD caused by autoimmune disease/glomerulonephritis/vasculitis is small especially in NDD-CKD, further investigation is needed before any conclusions can be drawn. In patients with HDD-CKD, males were independently associated with relatively better responsiveness to vadadustat, however the same result was not observed in NDD-CKD. The reason why sex difference was identified as a factor affecting the responsiveness to vadadustat only in patients with HDD-CKD remains unclear, and a more detailed examination should lead to a definitive conclusion.

In this post-hoc analysis, among the factors such as ESA hyporesponsiveness, low eGFR, and high CRP positively affected vadadustat responsiveness, while low ferritin levels were negatively related to vadadustat responsiveness. Although several factors affecting the vadadustat responsiveness were identified, the mean hemoglobin levels in each subgroup stratified by these factors were within the target range at weeks 20–24, with adjustment of vadadustat dose (150–600 mg).

The following limitations should be considered when interpreting the results of this study. This is a post-hoc analysis of the phase 3 studies (NCT03329196 and NCT03439137) conducted in patients with NDD and HDD-CKD, and the number of patients was not designed for this study; therefore, the results obtained from this analysis need to be validated. The sample sizes in some subgroups were small and may have been insufficient to obtain reliable results. Finally, it is not possible to investigate the confounding effects of patient background factors not obtained in the clinical trials.

## Conclusions

This post-hoc analysis of the Japanese phase 3 studies identified independent factors associated with a better response to vadadustat in anemia patients: high baseline hemoglobin, low baseline eGFR, high week-20–24 ferritin, and CKD not caused by autoimmune disease/glomerulonephritis/vasculitis in NDD-CKD and male sex, high baseline CRP, and low baseline ERI in HDD-CKD. These results would be expected to provide useful information leading to an appropriate dose modification for vadadustat.

### Supplementary Information

Below is the link to the electronic supplementary material.

Supplementary file1 (DOCX 253 KB)
