# Supplementary material for: Factors affecting responsiveness of vadadustat in patients with anemia associated with chronic kidney disease: a post-hoc subgroup analysis of Japanese phase 3 randomized studies
Source: Clin Exp Nephrol. 2024 Mar 26;28(5):391–403. doi: 10.1007/s10157-023-02432-z (PMC11033221; doi:10.1007/s10157-023-02432-z)
Supplement: Supplementary file 1 — Supplementary file1 (DOCX 253 KB) [file 10157_2023_2432_MOESM1_ESM.docx]

**Factors Affecting Responsiveness of Vadadustat in Patients with Anemia Associated with Chronic Kidney Disease: A Post-hoc Subgroup Analysis of Japanese Phase 3 Randomized Studies**

**Supplementary figures and tables**

**Authors:**

Masaomi NANGAKU, MD, PhD

Kiichiro UETA, PhD

Kenichi NISHIMURA

Kazuyo SASAKI

Takafumi HASHIMOTO

**
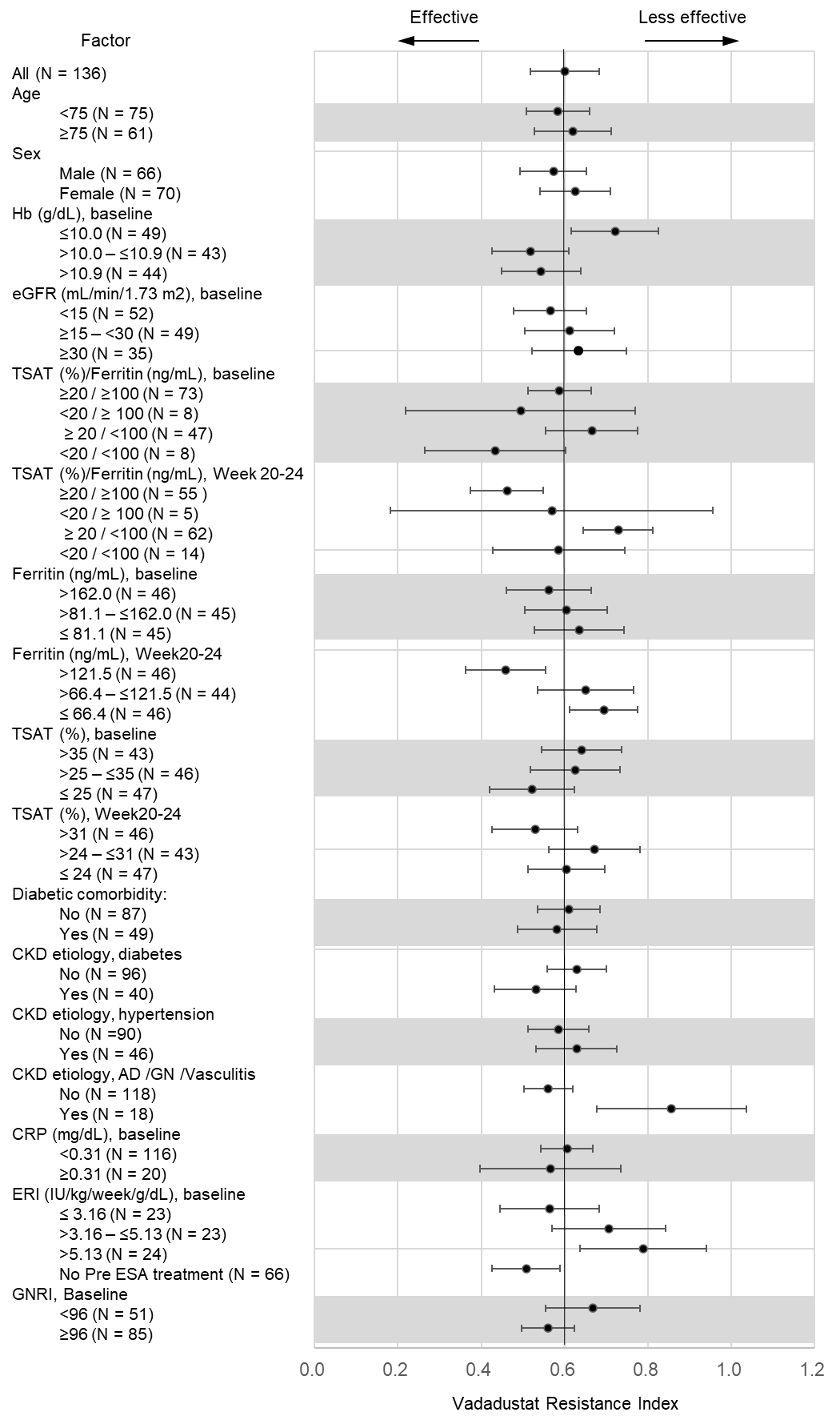
**

**Supplementary Figure 1: Forest plot of the vadadustat resistance index in nondialysis-dependent patients**

The vadadustat resistance index was defined as the mean weight-adjusted dose of vadadustat (dose/kg) at weeks 20–24 divided by the mean hemoglobin (g/dL) at weeks 20–24. The symbols and bars indicate the means and 95% confidence interval, respectively.


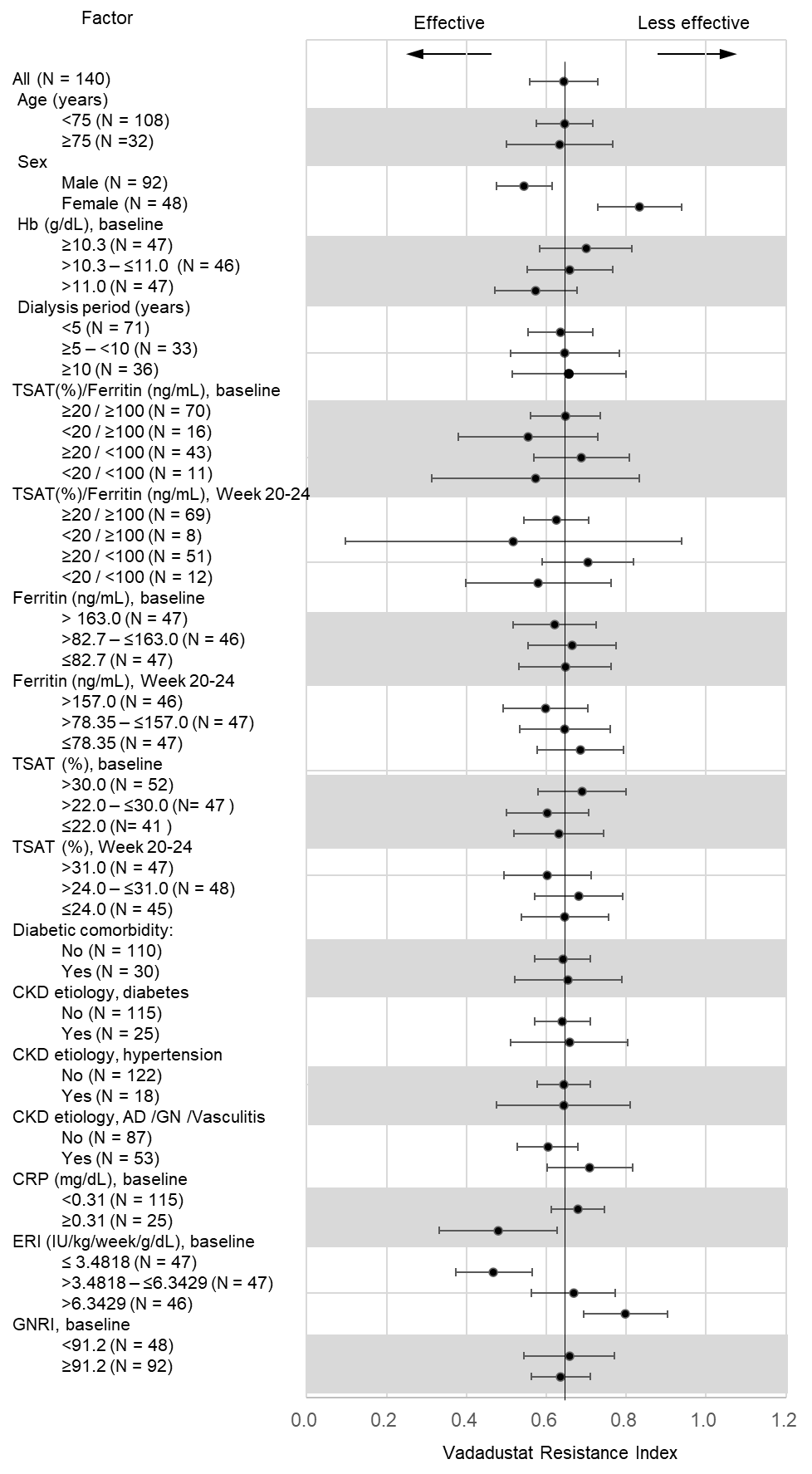


**Supplementary Figure 2: Forest plot of the vadadustat resistance index in hemodialysis-dependent patients**

The vadadustat resistance index was defined as the mean weight-adjusted dose of vadadustat (dose/kg) at weeks 20–24 divided by the mean hemoglobin (g/dL) at weeks 20–24. The symbols and bars indicate the means and 95% confidence interval, respectively.

**S****upplementary Table 1: Hemoglobin levels and dose of vadadustat at weeks 20-24 in patients with nondialysis-dependent CKD**

|  |  |  | Hb (g/dL) | | Dose (mg/day) | | |
| --- | --- | --- | --- | --- | --- | --- | --- |
| Factor | Category | N | Mean | 95% CI | Mean | 95% CI |  |
| All |  | 136 | 11.67 | 11.50, 11.84 | 379.26 | 347.53, 410.98 |  |
| Age [years] | <75 | 75 | 11.85 | 11.61, 12.10 | 389.71 | 345.64, 433.79 |  |
|  | ≥75 | 61 | 11.44 | 11.20, 11.69 | 366.40 | 319.75, 413.05 |  |
| Sex | Male | 66 | 11.51 | 11.25, 11.77 | 392.80 | 346.58, 439.02 |  |
|  | Female | 70 | 11.82 | 11.59, 12.05 | 366.49 | 321.99, 410.99 |  |
| Hb [g/dL], baseline | ≤10.0 | 49 | 11.34 | 10.98, 11.71 | 442.65 | 387.48, 497.82 |  |
|  | >10.0– ≤10.9 | 43 | 11.91 | 11.65, 12.16 | 347.84 | 291.58, 404.10 |  |
|  | >10.9 | 44 | 11.80 | 11.56, 12.04 | 339.36 | 287.71, 391.01 |  |
| eGFR [mL/min/1.73m^2^], baseline | <15 | 52 | 11.66 | 11.38, 11.93 | 364.60 | 311.27, 417.93 |  |
|  | ≥15 – <30 | 49 | 11.60 | 11.29, 11.91 | 377.95 | 324.42, 431.48 |  |
|  | ≥30 | 35 | 11.79 | 11.44, 12.14 | 402.86 | 339.54, 466.17 |  |
| Iron repletion (TSAT [%]/Ferritin [ng/mL], baseline) | ≥20/≥100 | 73 | 11.62 | 11.41, 11.82 | 372.36 | 330.74, 413.99 |  |
|  | <20/≥100 | 8 | 12.39 | 11.43, 13.36 | 355.58 | 179.76, 531.40 |  |
|  | ≥20/<100 | 47 | 11.54 | 11.18, 11.90 | 407.94 | 348.39, 467.49 |  |
|  | <20/<100 | 8 | 12.18 | 11.75, 12.61 | 297.32 | 183.04, 411.61 |  |
| Iron repletion (TSAT [%]/Ferritin [ng/mL], week 20-24) | ≥20/≥100 | 55 | 11.63 | 11.33, 11.94 | 285.40 | 241.21, 329.58 |  |
|  | <20/≥100 | 5 | 11.74 | 10.71, 12.77 | 358.93 | 148.90, 568.96 |  |
|  | ≥20/<100 | 62 | 11.64 | 11.38, 11.90 | 458.38 | 413.64, 503.13 |  |
|  | <20/<100 | 14 | 11.94 | 11.54, 12.35 | 404.85 | 304.89, 504.80 |  |
| Ferritin [ng/mL], baseline | >162.0 | 46 | 11.66 | 11.37, 11.95 | 350.99 | 296.24, 405.74 |  |
|  | >81.1 – ≤162.0 | 45 | 11.76 | 11.49, 12.03 | 389.20 | 336.47, 441.92 |  |
|  | ≤81.1 | 45 | 11.59 | 11.23, 11.95 | 398.21 | 337.75, 458.68 |  |
| Ferritin [ng/mL], week 20-24 | >121.5 | 46 | 11.56 | 11.23, 11.88 | 281.96 | 235.91, 328.01 |  |
|  | >66.4– ≤121.5 | 44 | 11.64 | 11.30, 11.98 | 383.77 | 323.79, 443.74 |  |
|  | ≤66.4 | 46 | 11.81 | 11.57, 12.06 | 472.24 | 425.09, 519.40 |  |
| TSAT [%], baseline | >35 | 43 | 11.39 | 11.12, 11.67 | 405.02 | 352.07, 457.98 |  |
|  | >25 – ≤35 | 46 | 11.78 | 11.47, 12.08 | 389.56 | 328.36, 450.75 |  |
|  | ≤25 | 47 | 11.82 | 11.50, 12.14 | 345.60 | 292.87, 398.33 |  |
| TSAT [%], week 20-24 | >31 | 46 | 11.61 | 11.28, 11.94 | 330.40 | 273.32, 387.48 |  |
|  | >24– ≤31 | 43 | 11.52 | 11.18, 11.85 | 407.77 | 349.32, 466.23 |  |
|  | ≤24 | 47 | 11.87 | 11.62, 12.12 | 400.99 | 350.14, 451.83 |  |
| Diabetic comorbidity | No | 87 | 11.61 | 11.38, 11.84 | 366.94 | 327.39, 406.49 |  |
|  | Yes | 49 | 11.77 | 11.51, 12.04 | 401.13 | 346.66, 455.60 |  |
| Cause of CKD: Diabetes | No | 96 | 11.57 | 11.35, 11.79 | 380.98 | 343.04, 418.91 |  |
|  | Yes | 40 | 11.90 | 11.62, 12.18 | 375.13 | 314.66, 435.60 |  |
| Cause of CKD: Hypertension | No | 90 | 11.74 | 11.52, 11.96 | 372.99 | 333.21, 412.77 |  |
|  | Yes | 46 | 11.53 | 11.24, 11.83 | 391.51 | 337.23, 445.79 |  |
| Cause of CKD: AD/GN/Vasculitis | No | 118 | 11.74 | 11.56, 11.93 | 363.65 | 329.56, 397.75 |  |
|  | Yes | 18 | 11.19 | 10.66, 11.72 | 481.55 | 403.87, 559.23 |  |
| CRP [mg/dL], baseline | <0.31 | 116 | 11.66 | 11.47, 11.85 | 382.67 | 348.07, 417.26 |  |
|  | ≥0.31 | 20 | 11.73 | 11.22, 12.25 | 359.46 | 273.16, 445.77 |  |
| ERI [IU/kg/week/g/dL], baseline | ≤3.16 | 23 | 11.59 | 11.18, 11.99 | 377.33 | 303.51, 451.15 |  |
|  | >3.16 – ≤5.13 | 23 | 11.48 | 11.00, 11.95 | 444.18 | 370.02, 518.34 |  |
|  | >5.13 | 24 | 11.30 | 10.88, 11.72 | 440.46 | 367.54, 513.39 |  |
|  | No Pre ESA | 66 | 11.90 | 11.65, 12.15 | 335.05 | 287.47, 382.62 |  |
| GNRI, baseline | <96 | 51 | 11.52 | 11.22, 11.81 | 364.61 | 309.69, 419.53 |  |
|  | ≥96 | 85 | 11.76 | 11.55, 11.98 | 388.05 | 348.66, 427.43 |  |

**S****upplementary Table 2: Hemoglobin levels and dose of vadadustat at weeks 20-24 in patients with hemodialysis-dependent CKD**

|  |  |  |  | | Hb (g/dL) | | Dose (mg/day) | |
| --- | --- | --- | --- | --- | --- | --- | --- | --- |
| Factor | Category | | | N | Mean | 95% CI | Mean | 95% CI |
| All |  | | | 140 | 10.66 | 10.52, 10.80 | 375.34 | 343.45, 407.23 |
| Age [years] | <75 | | | 108 | 10.62 | 10.45, 10.79 | 382.13 | 345.52, 418.75 |
|  | ≥75 | | | 32 | 10.79 | 10.51, 11.07 | 352.40 | 284.52, 420.28 |
| Sex | Male | | | 92 | 10.74 | 10.55, 10.92 | 344.49 | 304.42, 384.55 |
|  | Female | | | 48 | 10.52 | 10.29, 10.74 | 434.46 | 384.33, 484.60 |
| Hb [g/dL], baseline | ≤10.3 | | | 47 | 10.49 | 10.24, 10.74 | 391.96 | 337.39, 446.53 |
|  | >10.3– ≤11.0 | | | 46 | 10.71 | 10.46, 10.96 | 382.10 | 322.99, 441.22 |
|  | >11.0 | | | 47 | 10.78 | 10.52, 11.04 | 352.09 | 296.48, 407.70 |
| Dialysis period [years] | <5 | | | 71 | 10.67 | 10.48, 10.86 | 386.98 | 343.87, 430.09 |
|  | ≥5 – <10 | | | 33 | 10.67 | 10.37, 10.96 | 371.92 | 301.72, 442.11 |
|  | >10 | | | 36 | 10.64 | 10.30, 10.98 | 355.51 | 286.33, 424.68 |
| Iron repletion (TSAT[%]/Ferritin[ng/mL]), baseline | ≥20/≥100 | | | 70 | 10.58 | 10.40, 10.76 | 381.41 | 334.23, 428.59 |
|  | <20/≥100 | | | 16 | 11.15 | 10.49, 11.80 | 354.58 | 254.38, 454.77 |
|  | ≥20/<100 | | | 43 | 10.58 | 10.32, 10.84 | 382.48 | 325.65, 439.30 |
|  | <20/<100 | | | 11 | 10.82 | 10.33, 11.31 | 338.96 | 209.22, 468.71 |
| Iron repletion (TSAT[%]/Ferritin[ng/mL]), baseline | ≥20/≥100 | | | 69 | 10.62 | 10.45, 10.79 | 369.39 | 324.06, 414.73 |
|  | <20/≥100 | | | 8 | 10.65 | 9.35, 11.95 | 313.39 | 102.09, 524.70 |
|  | ≥20/<100 | | | 51 | 10.72 | 10.45, 10.98 | 395.38 | 341.21, 449.55 |
|  | <20/<100 | | | 12 | 10.66 | 10.17, 11.15 | 365.63 | 263.58, 467.67 |
| Ferritin [ng/mL], baseline | >163.0 | | | 47 | 10.61 | 10.38, 10.85 | 369.50 | 312.65, 426.36 |
|  | >82.7 – ≤163.0 | | | 46 | 10.75 | 10.47, 11.04 | 394.68 | 336.69, 452.68 |
|  | ≤82.7 | | | 47 | 10.62 | 10.38, 10.86 | 362.23 | 307.57, 416.90 |
| Ferritin [ng/mL], week 20-24 | >157.0 | | | 46 | 10.58 | 10.34, 10.82 | 356.60 | 296.47, 416.72 |
|  | >78.35 – ≥157.0 | | | 47 | 10.67 | 10.42, 10.92 | 369.62 | 313.80, 425.43 |
|  | ≤78.35 | | | 47 | 10.73 | 10.46, 11.01 | 399.39 | 346.18, 452.60 |
| TSAT [%], baseline | >30.0 | | | 52 | 10.49 | 10.25, 10.74 | 394.16 | 340.79, 447.54 |
|  | >22.0 – ≤30.0 | | | 47 | 10.66 | 10.45, 10.87 | 354.23 | 296.75, 411.71 |
|  | ≤22.0 | | | 41 | 10.87 | 10.57, 11.17 | 375.65 | 316.90, 434.41 |
| TSAT [%], week 20-24 | >31.0 | | | 47 | 10.57, | 10.34, 10.80 | 345.14 | 289.19, 401.09 |
|  | >24.0 – ≤31.0 | | | 48 | 10.68 | 10.45, 10.90 | 392.27 | 335.27, 449.28 |
|  | ≤24.0 | | | 45 | 10.74 | 10.43, 11.05 | 388.81 | 333.04, 444.58 |
| Diabetic comorbidity | No | | | 110 | 10.71 | 10.54, 10.87 | 364.23 | 328.56, 399.90 |
|  | Yes | | | 30 | 10.49 | 10.17, 10.81 | 416.07 | 342.82, 489.32 |
| Cause of CKD: Diabetes | No | | | 115 | 10.71 | 10.55, 10.88 | 365.35 | 330.54, 400.16 |
|  | Yes | | | 25 | 10.42 | 10.12, 10.72 | 421.29 | 339.16, 503.41 |
| Cause of CKD: Hypertension | No | | | 122 | 10.64 | 10.49, 10.80 | 372.27 | 337.99, 406.55 |
|  | Yes | | | 18 | 10.78 | 10.41, 11.14 | 396.13 | 300.52, 491.74 |
| Cause of CKD: AD/GN/Vasculitis | No | | | 87 | 10.70 | 10.52, 10.89 | 362.49 | 321.01, 403.96 |
|  | Yes | | | 53 | 10.59 | 10.36, 10.83 | 396.43 | 345.60, 447.26 |
| CRP [mg/dL], baseline | <0.31 | | | 115 | 10.58 | 10.44, 10.73 | 388.55 | 354.03, 423.06 |
|  | ≥0.31 | | | 25 | 11.02 | 10.59, 11.46 | 314.57 | 231.52, 397.62 |
| ERI [IU/kg/week/g/dL], baseline | ≤3.4818 | | | 47 | 10.90 | 10.62, 11.17 | 306.61 | 247.71, 365.52 |
|  | >3.4818 – ≤6.3429 | | | 47 | 10.63 | 10.40, 10.87 | 375.89 | 324.39, 427.38 |
|  | >6.3429 | | | 46 | 10.45 | 10.22, 10.69 | 444.99 | 393.54, 496.45 |
| GNRI, baseline | <91.2 | | | 48 | 10.70 | 10.45, 10.96 | 338.26 | 286.19, 390.33 |
|  | ≥91.2 | | | 92 | 10.64 | 10.46, 10.82 | 394.68 | 354.35, 435.01 |

**Supplementary Table 3: Hepcidin levels stratified by baseline eGFR category in patients with nondialysis-dependent CKD**

| eGFR [mL/min/1.73m^2^], baseline |  | N | Hepcidin (ng/mL) | |
| --- | --- | --- | --- | --- |
|  |  |  | Mean | 95% CI |
| <15 | Baseline | 50 | 79.516 | 64.665, 94.368 |
|  | Week 2 | 52 | 32.706 | 24.481, 40.931 |
|  | Week 4 | 52 | 31.079 | 22.282, 39.875 |
|  | Week 6 | 52 | 27.634 | 21.333, 33.936 |
|  | Week 12 | 52 | 24.516 | 18.571, 30.462 |
|  | Week 24 | 49 | 37.608 | 28.217, 46.999 |
| ≥15–<30 | Baseline | 49 | 50.194 | 42.757, 57.631 |
|  | Week 2 | 49 | 26.230 | 19.713, 32.747 |
|  | Week 4 | 49 | 23.892 | 19.064, 28.720 |
|  | Week 6 | 49 | 21.150 | 16.006, 26.293 |
|  | Week 12 | 49 | 20.993 | 15.600, 26.386 |
|  | Week 24 | 49 | 27.998 | 20.229, 35.767 |
| ≥30 | Baseline | 35 | 46.168 | 32.633, 59.703 |
|  | Week 2 | 35 | 40.557 | 23.566, 57.548 |
|  | Week 4 | 35 | 32.614 | 22.205, 43.023 |
|  | Week 6 | 35 | 27.200 | 17.252, 37.147 |
|  | Week 12 | 35 | 25.332 | 18.964, 31.700 |
|  | Week 24 | 34 | 26.830 | 19.716, 33.944 |

**Supplementary Table 4: Baseline CRP levels with or without autoimmune disease/glomerulonephritis/vasculitis of CKD etiology in patients with nondialysis-dependent or hemodialysis-dependent CKD**

|  | NDD-CKD | | | |  | HDD-CKD | | | |
| --- | --- | --- | --- | --- | --- | --- | --- | --- | --- |
| Autoimmune diseases or vasculitis | N | CRP (mg/dL) | | |  | N | CRP (mg/dL) | | |
|  |  | Mean | SD | Median |  |  | Mean | SD | Median |
| With | 18 | 0.257 | 0.492 | 0.035 |  | 53 | 0.366 | 1.459 | 0.040 |
| Without | 118 | 0.236 | 0.633 | 0.050 |  | 87 | 0.233 | 0.467 | 0.060 |

**Supplementary Table 5: Hepcidin levels stratified by baseline CRP category in patients with nondialysis-dependent or hemodialysis-dependent CKD**

|  |  | NDD-CKD | | |  | HDD-CKD | | |
| --- | --- | --- | --- | --- | --- | --- | --- | --- |
| CRP [mg/dL], baseline |  | N | Hepcidin (ng/mL) | |  | N | Hepcidin (ng/mL) | |
|  |  |  | Mean | 95% CI |  |  | Mean | 95% CI |
| <0.31 | Baseline | 115 | 57.528 | 50.076, 64.980 |  | 115 | 63.316 | 54.389, 72.243 |
|  | Week 2 | 116 | 31.443 | 26.357, 36.530 |  | 115 | 64.787 | 53.519, 76.055 |
|  | Week 4 | 116 | 29.376 | 24.449, 34.304 |  | 115 | 65.919 | 54.895, 76.943 |
|  | Week 6 | 116 | 24.215 | 20.593, 27.838 |  | 115 | 56.333 | 46.631, 66.035 |
|  | Week 12 | 116 | 23.441 | 19.700, 27.182 |  | 115 | 46.313 | 38.341, 54.285 |
|  | Week 24 | 112 | 32.211 | 26.795, 37.628 |  | 114 | 47.281 | 38.982, 55.579 |
| ≥0.31 | Baseline | 19 | 75.554 | 48.100, 103.008 |  | 25 | 80.800 | 56.800, 104.800 |
|  | Week 2 | 20 | 37.904 | 9.920, 65.887 |  | 25 | 82.811 | 52.651, 112.971 |
|  | Week 4 | 20 | 26.033 | 12.922, 39.145 |  | 25 | 86.917 | 58.474, 115.361 |
|  | Week 6 | 20 | 30.817 | 13.529, 48.104 |  | 25 | 70.331 | 46.843, 93.820 |
|  | Week 12 | 20 | 23.548 | 16.145, 30.950 |  | 25 | 56.536 | 37.446, 75.625 |
|  | Week 24 | 20 | 25.962 | 15.363, 36.562 |  | 24 | 54.461 | 36.113, 72.809 |
